# A Rare Complication of an Inflatable Penile Prosthesis: Penile Skin Necrosis Due to Inappropriate Use of the Condom Catheter

**DOI:** 10.7759/cureus.73911

**Published:** 2024-11-18

**Authors:** Umut Arslan, Burtaç Talha Akkurt, Serkan Akan

**Affiliations:** 1 Department of Urology, University of Health Sciences, Fatih Sultan Mehmet Training and Research Hospital, Istanbul, TUR

**Keywords:** condom catheter, gangrene of penis, iatrogenic complication, inflatable penile prosthesis, skin necrosis

## Abstract

Penile prosthesis implantation is considered a last-resort treatment for erectile dysfunction, used when pharmacological and other conservative treatments are inadequate or at the patient’s request. The well-documented complications of penile prostheses include pain, infection, mechanical failure, improper positioning, and erosion.

In this case, we report a patient presenting with penile skin necrosis, despite the absence of typical risk factors such as diabetes mellitus, atherosclerosis, or chronic renal failure, attributed to pressure from a condom catheter that was used 15 years after the inflatable penile prosthesis implantation.

## Introduction

Penile prosthesis implantation serves as an outstanding end-stage treatment option for patients with severe erectile dysfunction (ED) who have not benefited from less invasive interventions [1]. In 1973, Scott et al. introduced the first inflatable penile prosthesis (IPP), and it has since become a frequently performed procedure over the subsequent decades [2]. The well-documented complications of penile prostheses include pain, infection, mechanical failure, improper positioning, and erosion, for which appropriate standard treatments have been established. However, the occurrence of penile gangrene is exceedingly rare, and due to the limited number of reported cases, a standardized treatment approach has not been established. Acute ischemia of the penis has been observed only rarely because of the excellent collateral circulation reaching the perineum and lower abdomen [3]. In such instances, removal of the prosthesis is generally recommended [4]. 

The condom catheter is designed for continuous 24/7 use but should be changed every one to two days [5]. In patients who are using condom catheters, catheter-related necrosis is a complication that is rarely seen. More common severe complications with their long-term use include urinary tract infections in 40% of cases, and skin injuries such as inflammation and ulceration in 15% of cases [6].

This case report presents the management of penile skin necrosis in a patient without diabetes, atherosclerosis, or chronic renal failure, attributed to prolonged condom catheter use 15 years after the implantation of the inflatable penile prosthesis.

## Case presentation

A 73-year-old male patient presented to the emergency department with weakness, impaired oral intake, and speech disturbances. Cranial CT revealed no bleeding, but diffusion-weighted MRI showed infarction in the left temporo-occipital region and the right middle cerebral artery (MCA)-posterior cerebral artery (PCA) border zone. He had gout as a comorbid condition; however, there was no history of vascular diseases, diabetes, trauma, chronic renal failure, or smoking, which could contribute to the necrosis. The patient was diagnosed with ischemic stroke, and received acetylsalicylic acid (ASA) 325 mg, followed by 100 mg daily and enoxaparin (60 mg) in the Neurology Intensive Care Unit (ICU). The patient's coagulation factors and infectious parameters were within normal limits.

**Figure 1:**
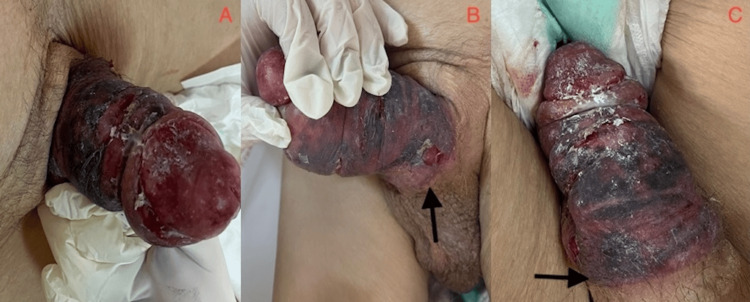
Preoperatively the area of gangrene was confined to the pressure zone of the condom catheter (black arrows)

On the third day post-admission, the transurethral Foley catheter placed in the emergency department was removed, and a condom catheter was initiated for urine output monitoring. On the seventh day, the patient developed ecchymosis on the penis and was referred to urology. An examination revealed an IPP that could not be detumesced. The patient exhibited elevated levels of C-reactive protein (CRP), measuring 158 mg/L (reference range 0-10 mg/L). The gangrenous area, characterized by coldness, swelling, and a foul odor, was confined to the pressure zone caused by the condom catheter (Figure 1).

**Figure 2:**
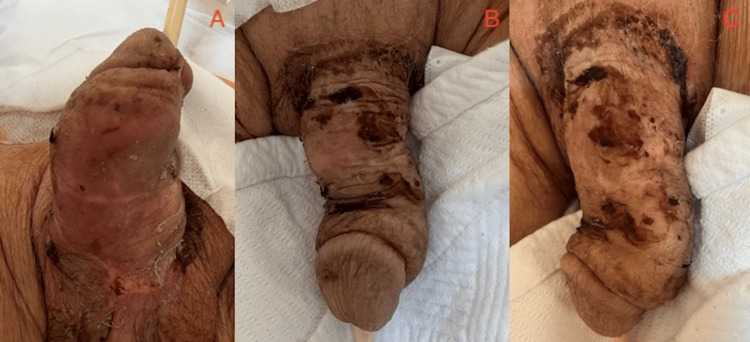
On day 10 after the operation, the previously gangrenous areas showed significant healing

We decided to remove the IPP, which had been implanted 15 years before, due to its mechanical malfunction that prevented detumescence. Initially, the cavernous cylinders and pump mechanism were removed through a penoscrotal incision. However, the reservoir could not be accessed through the penoscrotal region due to migration. A second incision was made in the right inguinal region to access it. The necrotic tissues in the penile skin and the subcutaneous layers were debrided, and primary repair was performed. No ischemia was observed in the Buck's fascia or the underlying tissues. A sandwich antibiotic dressing was applied, and the patient was transferred back to the ICU. Postoperatively, the patient was treated with intravenous meropenem 500 mg every eight hours and daily antibiotic wet dressings for 10 days (Figure 2).

## Discussion

Gangrene describes tissue necrosis and is typically classified into two types: dry gangrene, which results from reduced blood flow, and wet gangrene, which arises secondary to infection. Although the differentiation can be challenging, broad-spectrum antibiotics are generally recommended due to the likelihood of superinfection in the ischemic tissue [7]. Acute and chronic ischemia are major risk factors for gangrene formation. The blood supply to the skin of the penile shaft is primarily provided by the inferior external pudendal artery, while the internal pudendal artery branches into the urethral, cavernosal, and dorsal arteries [8]. In patients who have undergone IPP implantation, there is a potential for damage to the cavernosal arteries. This may lead to ischemia in the late postoperative period due to compression of the inferior external pudendal vessels by the condom catheter.

Penile ischemia and gangrene may result from IPP implantation, chronic renal failure, or advanced atherosclerosis. Other significant risk factors include priapism, pressure necrosis from dressings, vacuum devices, penile surgery, and most commonly, diabetes mellitus [9,10]. Contrary to the literature, our patient did not have diabetes or any other known risk factors but presented with iatrogenic ischemia that was diagnosed late and skin necrosis of the penile shaft due to internal and external pressure from the prosthesis and the condom catheter. Gangrene in the literature is typically observed on the penile glans and usually occurs within one month after the operation; however, in our case, it developed on the shaft skin 15 years after implantation due to the use of a condom catheter.

The condom catheter is typically used non-invasively in bedridden and incontinent patients. It is associated with fewer side effects compared to in-dwelling Foley catheters. Studies have demonstrated that condom catheters result in better outcomes in terms of pain, comfort, and urinary tract infections [11]. Despite a lower incidence of complications associated with condom catheters, they are not without risks, and a wide range of complications may occur. Some common ones include urinary tract infections, allergic reactions, inflammation, gangrene, and penile strangulation. Appropriate catheter sizing, changing the condom catheter every 48 hours, and performing checks during the change can help mitigate these risks [6]. 

The prevailing view is that prosthesis removal in cases of gangrene is crucial to prevent bacterial colonization and avoid extensive debridement or amputation [9]. Some surgeons may opt to retain an uninfected reservoir. In one study, 98 retained reservoirs were followed up for an average of 50 months without any cases of infection or erosion [12]. However, in our case, due to the malfunction of the inflatable penile prosthesis along with infection and gangrene, and considering the patient's advanced age and comorbidities, we decided not to leave any foreign body behind. Since access to the reservoir through the penoscrotal incision was not feasible, a larger right inguinal oblique incision was performed for removal. The literature supports the need for a second incision to remove the reservoir when its reuse is not anticipated or inguinal access is not achievable [13].

## Conclusions

Although the condom catheter is a non-invasive device, it carries a risk of penile complications, particularly in patients with a history of penile prosthesis implantation. Its prolonged and unnecessary use should be avoided in such cases. Patients without type 2 diabetes mellitus or peripheral arterial disease may still be susceptible to gangrene and infection due to IPP malfunction and the pressure exerted by a condom catheter, even in the late postoperative period. Regular follow-up, suitably-sized catheters, and appropriate duration of catheter use are recommended to mitigate these risks. In cases of complications, timely discussion with the patient or their surrogate regarding prosthesis removal and, if indicated, debridement is essential.
